# The influence of prior awareness on views about psychiatric electroceutical interventions among non-clinician stakeholders

**DOI:** 10.1038/s44184-023-00028-9

**Published:** 2023-05-03

**Authors:** J. M. Tyron, R. Bluhm, E. D. Achtyes, A. M. McCright, L. Y. Cabrera

**Affiliations:** 1 Pine Rest Christian Mental Health Services, Grand Rapids, MI 49548 USA; 2 Lyman Briggs and Philosophy, Michigan State University, East Lansing, MI 48824 USA; 3 Division of Psychiatry & Behavioral Medicine, Michigan State University, Grand Rapids, MI 49503 USA; 4 Sociology, Michigan State University, East Lansing, MI 48824 USA; 5 Center for Neural Engineering, Department of Engineering Science and Mechanics, Rock Ethics Institute, and Huck Institute of Life Sciences, Pennsylvania State University, University Park, PA 16802 USA

**Keywords:** Society, Medical ethics, Depression

## Abstract

Psychiatric electroceutical interventions (PEIs) are emerging interventions in the treatment of depression and other mood disorders. The uptake of PEIs is strongly influenced by public, caregiver, and patient views. This study examines the influence of prior awareness and of trust in the medico-scientific establishment with respect to non-clinicians’ views on PEI among a cohort of U.S. respondents. About 3098 U.S. caregivers, patients, and members of the general public completed an online survey with an embedded experiment to evaluate PEI views by stakeholder, modality, and disease severity. ANOVA statistical tests and logistic regression models were used to identify significant differences between groups and moderating factors. Overall, respondents had greater awareness of antidepressant medication (73–84%) and psychotherapy (68–76%) than of any PEI, and ECT garnered the most prior awareness (29–40%) within each group. Non-clinical respondents most often used websites or social media as information sources, and the least trusted sources included those with notable financial interests. Considering the low awareness level among non-clinicians, the implementation of programs to target and advance awareness levels about the use of PEIs in depression among this population may contribute to reducing negative views around these interventions. Fostering trust in the medico-scientific establishment may also increase public support for PEIs as well as uptake of these treatment modalities.

## Introduction

Over 20% of U.S. adults experienced mental illness in 2020. Untreated mental illness has far-reaching impacts, such as increased hospitalizations and emergency room visits, increased rates of employment and disability, and increased likelihood of developing comorbidities such as substance use disorders (https://www.nami.org/mhstats). Psychiatric electroceutical interventions (PEIs) have been options for treating mental illnesses for decades since electroconvulsive therapy (ECT) was first developed in 1938^1,2^. ECT is considered one of the most efficacious psychiatric treatments due to its speed and magnitude of response, safety, and cost effectiveness^2–7^.

More recent PEIs include repetitive transcranial magnetic stimulation (rTMS) and deep brain stimulation (DBS). rTMS, which uses a magnetic field to stimulate targeted cortical brain regions, has been FDA-approved for treating depression since 2008 and is currently being investigated for use in other disorders. rTMS is considered a non-invasive, safe, and evidence-based treatment for treatment-resistant depression (TRD) in adults^8–10^. Deep brain stimulation (DBS), which involves the surgical implantation of electrodes within specific brain regions, is considered the riskiest of the PEIs, primarily due to the invasiveness of the surgical procedure^11,12^. Nevertheless, DBS is FDA-approved for treating Parkinson’s disease, epilepsy, and other movement disorders^12,13^. Scientists are conducting early phase trials to determine its efficacy in treating psychiatric disorders such as TRD, but research supporting its use in TRD remains limited at this time^11–14^. A newer, though investigational, application of PEIs is adaptive brain implant (ABI) technology, which adjusts neurostimulation levels in response to a patient’s brain activity^15^.

Despite their clear efficacy, PEIs that are FDA-approved for treating mood disorders (such as ECT and rTMS) remain under-utilized^8,10,16^. Such under-utilization, at least in part, may be due to limited awareness of and/or negative attitudes about PEIs from non-clinicians (e.g., depressed patients, their caregivers, and the general public). Starting with Freeman and Kendall’s^17^ seminal work on attitudes toward ECT, much research analyzes how such attitudes influence the utilization of PEI treatments, especially ECT^18–20^. Assessing attitudes toward PEIs is important, as they may determine the public acceptability of a treatment, which in turn may influence help-seeking behaviors and ultimately demand a particular treatment^19,21^. Patients who are considering PEIs often rely on relatives and caregivers to inform their decision about treatments, so assessing caregivers’ perceptions is also important^22^.

Research shows persistent negative perceptions of ECT by non-clinicians, particularly among the general public. Griffiths and O’Neill-Kerr^22^ described how the public reports negative views of ECT in the face of media portrayals of ECT as inhumane, barbaric, punitive, and mind-altering—even though such portrayals do not accurately reflect current practice, patient experiences, and expected outcomes^18,20–26^. Despite negative portrayals in mass media, many studies have shown satisfaction with and positive attitudes toward ECT by patients who have received ECT and their families, even with reports of subjective memory impairment^20,27^. Rose notes that non-clinician perceptions and attitudes regarding ECT are complex and that this complexity is not always captured in the literature. Results of studies of non-clinicians’ perceptions of ECT vary widely and are influenced by study design, length and complexity of survey questions, the timing of when the study was conducted (e.g., length of time after treatment), as well as the source of the research (e.g., patient vs. clinician-led studies)^20^.

Much less research has been done examining public views of newer PEI modalities such as rTMS, DBS, or ABIs. Some work suggests that people who lack specific knowledge of newer PEI interventions may rely on general attitudes toward more well-known PEIs (like ECT), which are largely negative^8^. In the absence of reliable information, negative attitudes toward PEIs proliferate and influence perceptions of these treatments’ efficacy, safety, and side effects^1,8,21,25^.

These studies illustrate the need for more research on non-clinicians’ awareness of PEIs and how this factor may influence their attitudes toward PEIs. Further, in the absence of direct experience with these PEIs, non-clinicians’ views about them may be shaped more broadly by their general trust in medical professionals and the scientific community. While Vale and Good^28^ noted a general decline in such trust among US adults over the past several decades, existing research on the impact of trust in the medico-scientific establishment is limited. This study examines the influence of prior awareness of PEIs and trust in the medico-scientific establishment on views about PEIs among three US samples of non-clinicians: depressed patients, caregivers, and the general public. This study addresses the following research questions about these three non-clinician stakeholder groups:

1. How do PEI views (affect, influence, benefit, risk, and invasiveness) vary by stakeholders’ prior awareness of PEIs?
2. How do PEI views (affect, influence, benefit, risk, and invasiveness) vary by trust in the medico-scientific establishment?
3. Does the influence of (a) prior awareness of a PEI or (b) trust in the medico-scientific establishment on PEI views vary by stakeholder group, PEI modality, or TRD severity?

## Methods

### Study design

A standardized online survey with an embedded video vignette experiment was given to four large U.S. samples of the general public, caregivers, depressed patients, and board-certified psychiatrists. A between-subjects full factorial design was employed, which scholars have used in several fields^29–32^. Crossing the two factors—intervention type (ECT vs. rTMS vs. DBS vs. ABIs) and depression severity (moderate vs. severe depression)—produced eight total conditions in the video vignette. Each respondent was randomly assigned to one of these experimental conditions. All participants received the same set of core questions, in addition to a few unique to each stakeholder group. The current study focuses only on non-clinicians’ responses to five novel scales measuring key ethical concerns, beliefs, and attitudes about PEIs and to questions measuring prior PEI awareness, sources of mental health information, and perceived trust in such information sources.

After securing a human subjects exemption from a University Institutional Review Board (STUDY00001247), researchers contracted with online panel provider Qualtrics to recruit participants and administer the survey. The full survey was administered between April and June 2020 and is located in the Supplementary Methods.

### Participants

Qualtrics manages a large internet panel designed to capture the demographic diversity of the U.S. adult population and has the capacity to generate additional online panels of more specific subgroups within the adult population. Researchers contracted with Qualtrics to draw samples of U.S. adults to represent three non-clinician stakeholder groups. Table 1 below displays key social and demographic characteristics for each of the three samples in this study.

**Table 1:** Demographic, social, and political characteristics of three non-clinician stakeholder samples.

Characteristic	2020 US adult population estimates	General public sample (*N* = 1022)	Caregiver sample (*N* = 1026)	Depressed patient sample (*N* = 1050)
% Female	50.8^a^	50.9	64.2	73.7
Age				
% 18–24	12.1^b^	12.8	15.2	23.3
% 25–34	18.6^b^	17.7	21.4	20.6
% 35–44	17.0^b^	16.7	25.4	21.7
% 45–54	16.3^b^	17.5	16.1	17.7
% 55–64	17.1^b^	16.3	12.3	12.8
% 65 or older	22.4^b^	18.9	9.6	3.9
% White	76.3^a^	78.0	80.5	88.7
% Latinx	18.5^a^	17.4	13.3	8.3
% with Bachelor’s degree or higher	32.1^a^	45.2	54.6	33.3
Median household income				
% <$25,000	18.1^c^	17.5	12.5	
% $25,000–$49,999	19.7^c^	22.1	23.4	
% $50,000–$74,999	16.5^c^	19.7	19.2	
% $75,000–$99,999	12.2^c^	13.5	14.7	
% $100,000–$149,999	15.3^c^	15.1	15.9	
% $150,000–$199,999	8.0^c^	6.1	7.9	
% >$200,000	10.3^c^	6.1	6.4	
Political ideology				
% Conservative	34^d^	33.7	32.7	28.7
% Middle-of-the-road	36^d^	31.2	27.3	31.5
% Liberal	26^d^	35.2	40.0	39.8
Church attendance frequency				
% Never	25.6^e^	34.8	27.9	40.6
% About once a year	24.3^e^	12.2	9.8	12.7
% A few times a year	16.5^e^	17.9	17.3	15.9
% Once a month	7.9^e^	3.6	5.9	4.8
% A few times a month	18.0^e^	10.3	12.7	7.7
% At least once every week	25.3^e^	21.1	26.3	18.4

The following sources were used to obtain the 2020 US Population Estimates:

^a^https://www.census.gov/quickfacts/fact/table/US/PST045221.

^b^https://www.statista.com/statistics/241488/population-of-the-us-by-sex-and-age/.

^c^https://www.census.gov/data/tables/time-series/demo/income-poverty/cps-hinc/hinc-01.html.

^d^https://news.gallup.com/poll/316094/conservatism-down-start-2020.aspx.

^e^https://www.pewforum.org/dataset/american-trends-panel-wave-61/.

For the general public stakeholder group, Qualtrics drew a sample of 1022 adults from its primary internet panel that closely matches spring 2020 U.S. population estimates of sex, age, race, ethnicity, educational attainment, income, political ideology, and religiosity. For the caregiver stakeholder group, Qualtrics applied a screening question with its primary internet panel to select 1026 adults who were currently serving as the primary caregiver for a family member or friend with depression. For the patient stakeholder group, Qualtrics drew a sample of 1050 adults with depression from a separate internet panel of adults who had previously reported a depression diagnosis. This sample of depressed patients aligns with age, sex, and race estimates of the U.S. adult population living with depression in 2020 (https://www.nimh.nih.gov/health/statistics/major-depression).

### Procedures

The first page of the survey provided participants with information about the study and their participation, and they indicated their consent to participate by clicking through to the rest of the survey. Participants then answered a set of questions about their awareness of different psychiatric interventions, sources of information about psychiatric treatments, and trust in such information sources. Then participants viewed a randomly assigned video vignette—featuring professional actors playing a patient with moderate or severe TRD receiving information about one of the four PEIs from a psychiatrist^33–37^. After answering initial questions assessing participant understanding of the experimental message, participants answered several questions measuring key views about the PEI featured in their video as well as their demographic, social, and political characteristics^38^. The privacy rights of participants were protected throughout the course of the study.

### Measures

Because there are no specific measures of views about PEIs, the research team created several novel instruments to measure key PEI views through a multi-stage process^39^. Informed by the results of a principal components analysis (see Supplementary Table A) and reliability analysis, researchers produced the following five scales:

- an 8-item *General Affect Scale* (Cronbach’s α = 0.93), ranging from “negative” (1) to “positive” (7);
- a 7-item *Perceived Influence on Self Scale* (Cronbach’s α = 0.94), ranging from “strong negative influence” (1) to “strong positive influence” (7);
- a 6-item *Perceived Benefits Scale* (Cronbach’s α = 0.87), ranging from “no benefit at all” (1) to “great benefit” (6);
- a 5-item *Perceived Riskiness Scale* (Cronbach’s α = 0.87), ranging from “no risk at all” (1) to “great risk” (6); and
- a 6-item *Perceived Invasiveness Scale* (Cronbach’s α = 0.90), ranging from “not interfere at all” (1) to “greatly interfere” (6).

For each of these scales, item order was randomized to eliminate question order effects^40^.

**Fig. 1:**
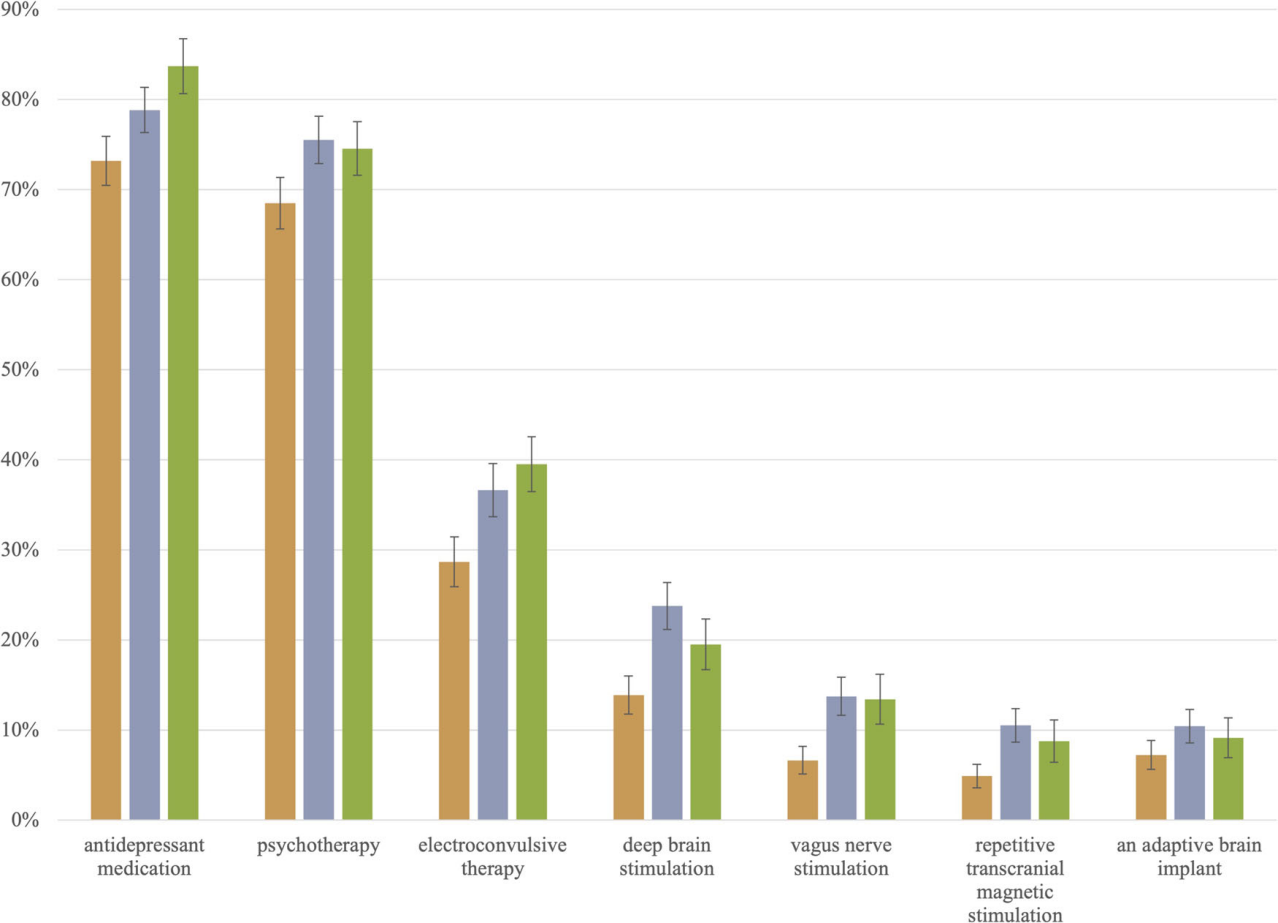
The percentage of each non-clinician group that reported prior awareness of selected interventions for depression. The three columns represent percentages for the general public (), caregivers (), and depressed patients (). The whisker/error bar on each column represents the 95% confidence interval.

**Fig. 2:**
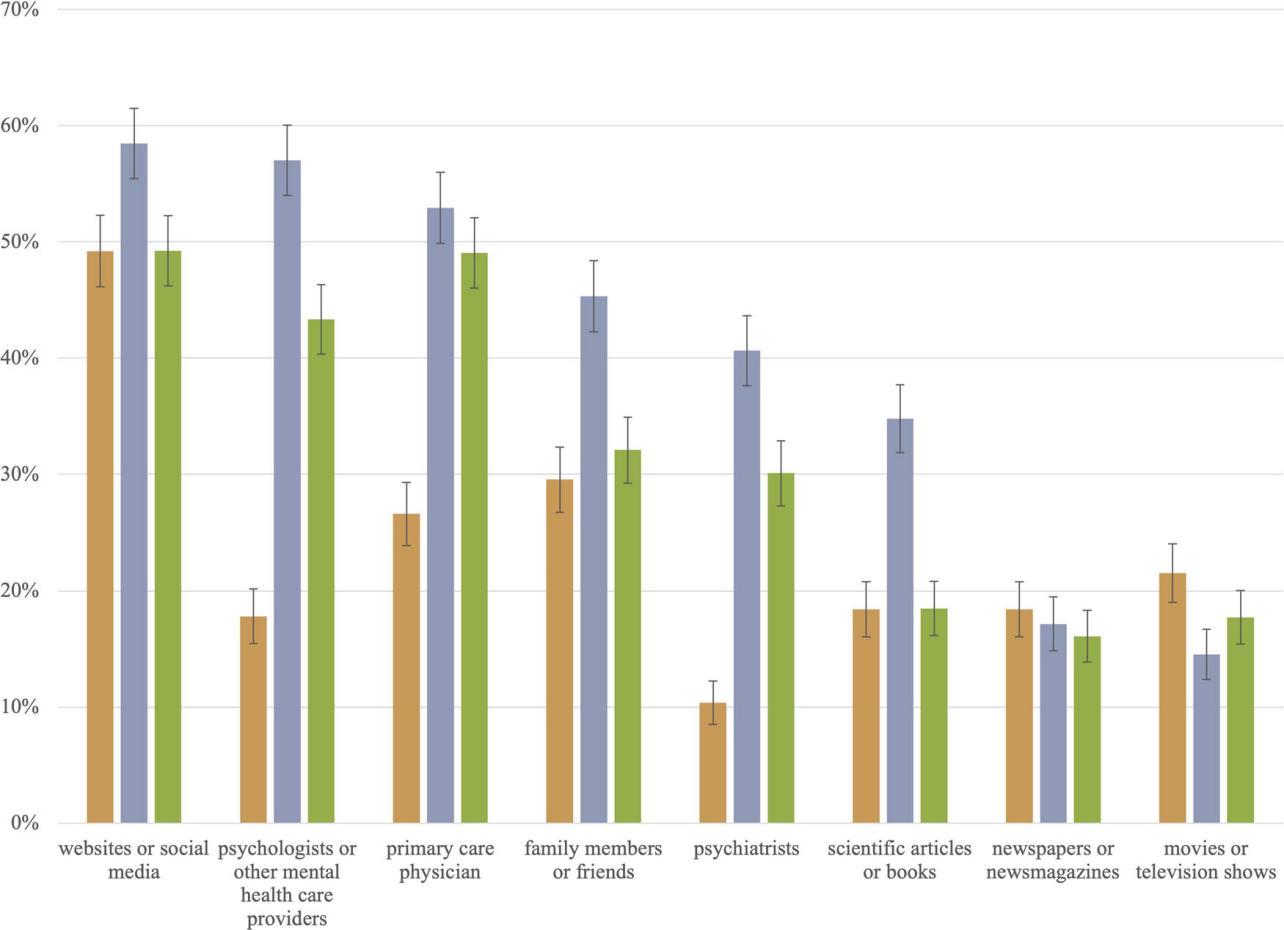
The percentage of each non-clinician group that receives mental health information from each selected source. The three columns represent percentages for the general public (), caregivers (), and depressed patients (). The whisker/error bar on each column represents the 95% confidence interval.

**Fig. 3:**
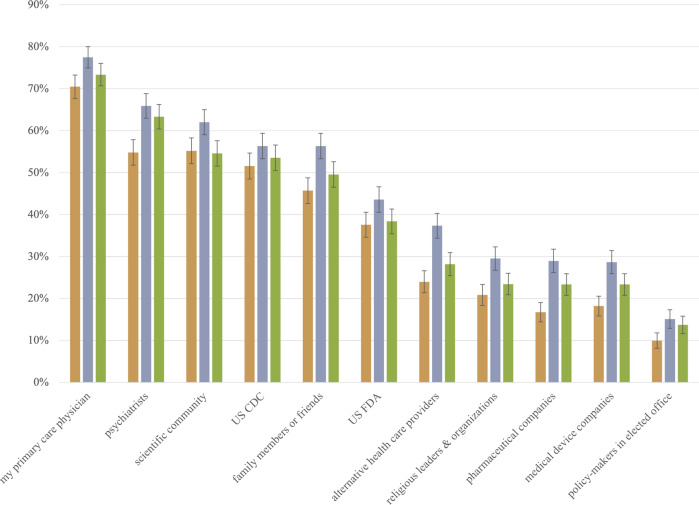
The percentage of each non-clinician group reporting moderate or strong trust in selected mental health information sources. The three columns represent percentages for the general public (), caregivers (), and depressed patients (). The whisker/error bar on each column represents the 95% confidence interval.

Participants reported their pre-survey awareness of the seven psychiatric interventions listed in Fig. 1 below. The dichotomous *prior awareness* indicates whether or not they were aware of their assigned PEI prior to the survey (“no”=0, “yes”=1). Participants also reported whether or not they received mental health information in the last year from any of the eight sources listed in Fig. 2 below. Participants also reported their level of distrust or trust in mental health information provided by each of the 11 sources listed in Fig. 3 below. A *trust in medico-scientific establishment* scale (Cronbach’s α = 0.81), ranging from “strongly distrust”=1 to “strongly trust”=7, measured the extent to which participants distrust or trust their primary care physician, psychiatrists, the scientific community, the U.S. Centers for Disease Control and Prevention (CDC), and the U.S. Food and Drug Administration (FDA). Further, participants’ perception of how bad it would be to live with TRD every day was measured with a single item (*bad daily life with TRD*) ranging from “moderately bad” (1) to “extremely bad” (10).

Finally, six demographic, social, and political characteristics employed as controls in statistical analyses were measured. Sex (“male”=0; “*female*”=1) and race (“non-white”=0; “*white*”=1) were measured with dummy variables. *Age* varied from “18–24” (1) to “65 or over” (6), and *educational attainment* varied from “high school diploma or GED” (1) to “graduate degree” (4). *Political ideology* was measured along a unidimensional scale from “very conservative” (1) to “very liberal” (7), and *religiosity* was measured as the frequency of religious service attendance, ranging from “never” (1) to “at least once every week” (6).

### Analytical techniques

Analyses were conducted in three stages with IBM SPSS 26.0. First, a series of one-way ANOVAs were performed to examine the differences in means among non-clinicians’ (a) prior awareness of psychiatric treatments, (b) sources of mental health information over the last 12 months, and (c) level of trust in selected mental health information sources. Second, to answer RQ1 and RQ2, a series of multiple ordinary least squares (OLS) regression models were completed to examine variation in PEI views by stakeholder’s *prior awareness* of their assigned PEI and *trust in the medico-scientific establishment* while accounting for respondents’ demographic, social, and political characteristics. The main effects of the experimental factors (i.e., PEI modality and depression severity) and stakeholder group membership (i.e., patients, caregivers, and members of the public) were modeled with dummy variables, with ECT modality, moderate depression, and the general public as the reference groups, respectively. Third, to answer RQ3, researchers included interaction terms in these multiple OLS regression models to examine whether the influence of *prior PEI awareness* or *trust in the medico-scientific establishment* on PEIs views vary by stakeholder group membership, PEI modality, or depression severity. To reduce the likelihood of multicollinearity problems associated with using higher-order (e.g., interaction) terms in regression models, interaction terms were created using centered scores^41^. For balanced Likert-style items (i.e., bivalent items with a neutral middle category), missing values were recoded to the item’s middle/neutral category. For other items, missing values were recoded to the item’s median value.

## Results

The percentages and 95% confidence intervals in Figs. 1, 2, and 3 below visualize results from 26 one-way ANOVA models explaining prior awareness of depression interventions, sources of mental health information, and trust in mental health information sources by non-clinician stakeholder groups. Supplementary Table B displays these one-way ANOVA results. The *F*-statistic for each of the 26 models is statistically significant at *p* < 0.05, with only two exceptions. The percentages of non-clinicians receiving mental health information from newspapers or newsmagazines are not statistically different; neither are the percentages of non-clinicians reporting moderate or strong trust in the U.S. CDC.

### Prior awareness of PEIs

Three patterns regarding prior awareness of seven depression interventions are noteworthy (Fig. 1). First, greater percentages of each stakeholder group reported prior awareness of antidepressant medication (73–84%) and psychotherapy (68–76%) than of any PEI. Second, among the PEIs, ECT garnered the most prior awareness (29–40%) within each stakeholder group. Less than 25% of each stakeholder group reported prior awareness of rTMS or of any of the implantable PEIs. Third, for six conditions (all but ABIs), lower percentages of the general public reported prior awareness than did caregivers and patients.

### Trust in mental health information sources

Three patterns among respondents’ mental health information sources are noteworthy (Fig. 2). First, non-clinicians most often used information source is “new” media (i.e., websites or social media), and their least often used information sources are conventional media (i.e., newspapers or newsmagazines and movies or television shows). Second, compared to both caregivers and patients, much lower percentages of the general public reported getting mental health information from psychiatrists (10%), psychologists (18%), and primary care physicians (27%). Third, compared to patients, much higher percentages of caregivers reported getting mental health information from websites or social media (58% vs. 49% for patients), psychologists (57% vs. 43%), family or friends (45% vs. 32%), psychiatrists (41% vs. 30%), and scientific articles or books (35% vs. 18%).

Three patterns are noteworthy regarding non-clinician stakeholder groups reporting moderate or strong trust in 11 different mental health information sources (Fig. 3). First, four components of the medico-scientific establishment (primary care physicians, psychiatrists, the scientific community, and the U.S. CDC) each garnered moderate or strong trust from at least 50% of each group. Second, non-clinicians’ least trusted sources are those with notable financial interests (medical device companies and pharmaceutical companies) or ideational interests (policy-makers in elected offices and religious leaders and organizations) that may make them less trustworthy on matters of health and science. Third, compared to the other two groups, greater percentages of caregivers reported trust in sources beyond the medico-scientific establishment (e.g., family and friends, alternative healthcare providers, and religious leaders and organizations).

### PEI views by PEI prior awareness and trust in the medico-scientific establishment

The results in Table 2 provide answers for RQ1 and RQ2 about how non-clinicians’ PEI views vary by their prior PEI awareness and their trust in the medico-scientific establishment, respectively. Prior PEI awareness was positively associated with affect toward the same PEI but was not related to any other PEI view. Trust in the medico-scientific establishment was positively associated with three positive PEI views (i.e., affect toward PEI, perceived positive PEI influence on self, and perceived PEI benefit)—and also with perceived PEI invasiveness.

**Table 2:** Standardized coefficients from multiple OLS regression models explaining PEI views among non-clinicians (*N* = 3098).

Predictors	Affect toward PEI	Perceived Influence of PEI	Perceived benefit of PEI	Perceived risk of PEI	Perceived invasiveness of PEI
Stakeholders					
Caregivers (ref: public)	0.05*	0.02	0.05*	0.03	0.03
Patients (ref: public)	0.06**	0.04	0.05*	0.02	0.01
Experimental conditions					
rTMS (ref: ECT)	0.18***	0.09***	0.08***	−0.13***	−0.04
DBS (ref: ECT)	0.02	0.06**	0.05*	0.03	0.06**
ABI (ref: ECT)	−0.00	0.07**	0.05*	0.06**	0.07**
Severe TRD (ref: moderate)	0.04*	0.07***	0.03	0.02	0.03
Perception of TRD					
Bad daily life with TRD	−0.04*	0.08***	0.16***	−0.03	−0.02
Key mental health views					
Trust in medico-scientific establishment	0.19***	0.24***	0.22***	−0.00	0.05**
Prior PEI awareness	0.08***	0.03	0.01	0.02	0.01
Socio-demographics					
Female	−0.09***	0.02	−0.03	−0.09***	−0.07***
Age	−0.05**	−0.02	−0.04*	−0.23***	−0.21***
White	0.02	0.05**	0.02	−0.05**	−0.03
Educational attainment	0.10***	0.04*	0.03	0.00	0.02
Political ideology	−0.02	−0.02	−0.01	−0.01	−0.02
Religiosity	0.10***	0.05*	0.07***	0.06**	0.11***
**Adjusted** ***R***^**2**^	0.13	0.09	0.10	0.09	0.08

*p < 0.05, **p < 0.01, ***p < 0.001.

### PEI views by stakeholder group, PEI modality, and TRD severity

Results from multiple OLS regression models provide evidence that non-clinicians’ ethical concerns, beliefs, and attitudes vary only minimally across stakeholder groups and by depression severity and vary more considerably by PEI modality (Table 2). These models also account for participants’ perception of how bad it would be to live every day with TRD, their trust in the medico-scientific establishment, and their prior awareness of their assigned PEI—while controlling for key demographic, social, and political characteristics. For space and flow reasons, researchers placed the discussion of the performance of the demographic, social, and political control variables in the Supplementary discussion.

Compared to their counterparts in the general public, caregivers and patients reported slightly more positive affect toward and perceived benefit from their assigned PEI. Compared to those in the ECT condition, participants in the rTMS condition reported more strongly positive views and less strongly negative views toward their assigned PEI. Participants in the two implantable conditions (DBS and ABIs) reported more strongly positive views toward their assigned PEI than did their counterparts in the ECT condition; further, the former also perceived these implantable PEIs as more invasive than the latter perceived ECT. Also, compared to participants in the moderate depression condition, those in the severe depression condition reported more positive affect and more positive influence on self. Finally, the worse that participants perceived life with TRD, the more positive influence on self and the more positive benefit they perceived from their assigned PEI—but also slightly more negative affect toward their PEI.

### Influence of prior awareness and trust on PEI views by stakeholder group, PEI modality, and depression severity

RQ3 asks whether the influence of prior PEI awareness or trust in the medico-scientific establishment on PEI views varies by stakeholder group, PEI modality, or TRD severity. Researchers included the requisite interaction terms to the five models in Table 2 to test for these potential moderating effects. Supplementary Table C reports these expanded OLS regression models. Briefly, the stakeholder group does not moderate the influence of prior PEI awareness or the influence of trust in the medico-scientific establishment on PEI views—with one exception. Trust in the medico-scientific establishment is positively associated with perceived PEI invasiveness, but only among caregivers and patients. While PEI modality does not moderate the influence of trust in the medico-scientific establishment on PEI views, the influence of prior PEI awareness on PEI views does vary by PEI modality. For instance, prior PEI awareness is positively associated with positive affect toward that same PEI, but only among those in one of the implantable PEI conditions. Prior PEI awareness is positively associated with perceived PEI benefit, but only among those in the rTMS and DBS conditions. Further, prior PEI awareness is associated with greater perceived risk and greater perceived invasiveness, but only for participants in the ABI condition. Finally, depression severity moderates neither the influence of prior awareness nor the influence of trust on any PEI views.

## Discussion

These results provide insights about the under-utilization of PEIs that are FDA-approved for treating depression (i.e., ECT and rTMS) and the potential limitations to the future adoption of PEIs that are under investigation for treating depression (i.e., DBS and ABIs). Compared to first-line depression treatments, non-clinicians reported considerably less prior awareness of any PEI in this study—a pattern similar to that in other studies^8,26,42–44^. Yet, with the exception of a small positive influence on perceived affect, which corresponds with earlier results from Griffiths and O’Neill-Kerr^22^ and Tsai et al.^2^, prior PEI awareness was not associated with any other PEI views. This latter, stronger pattern parallels findings of other studies that (positive) PEI views are unrelated to PEI awareness^1,45^. Thus, to answer RQ1, non-clinicians report relatively low levels of PEI awareness, and—for the most part—non-clinicians’ prior PEI awareness is not associated with their attitudes and beliefs about these same PEIs.

Compared to other non-clinicians, the general public employed the narrowest approach to mental health information seeking, disproportionately relying upon social media and websites, which mirrors results from other studies^44^. At the same time, caregivers—likely due to the pressures of their caregiving responsibilities—employed the broadest approach, seeking mental health information from a wide array of sources. Further, even though each stakeholder group reported at least moderate trust in several components of the medico-scientific establishment, caregivers also reported substantial levels of trust in non-scientific sources. Given the pervasiveness of online misinformation (often shared by non-scientific sources) that portrays PEIs in a rather negative manner^46^, mental health communicators should prioritize efforts to counter any PEI misinformation that non-clinicians may find online.

While trust in mental health professionals, the scientific community, and relevant federal agencies (referred to here as the medico-scientific establishment) had a relatively weak positive association with perceived invasiveness, it had a stronger positive effect on perceived affect, perceived influence on self, and perceived benefit. Indeed, this trust measure is the strongest predictor in each of these three models. Thus, to answer RQ2, trust in the medico-scientific establishment is correlated substantially with important PEI views, supporting the findings of earlier studies^47,48^. Strengthening non-clinicians’ trust in the medico-scientific establishment may be key for cultivating public support for PEIs as well as uptake of these interventions.

While non-clinicians’ PEI views differed only minimally across stakeholder groups and by depression severity, they do vary considerably by PEI modality. For the most part, non-clinicians viewed rTMS, DBS, and ABIs more positively than ECT, which is consistent with earlier studies documenting rather negative public perceptions of ECT^8,26,45,49,50^ and lesser acceptability of ECT than of other treatments^21,51,52^. Further, non-clinicians viewed the two implantable PEIs (i.e., DBS and ABIs) to be more invasive than ECT. Briefly, this greater perceived invasiveness is likely influenced by concerns about surgery complications and unease with the idea of a foreign object in the brain, which feature prominently in prior studies^12,13^.

Finally, the third research question asked whether stakeholder group membership, depression severity, or PEI modality moderated the relationships between prior PEI awareness or trust in the medico-scientific establishment and PEI views. Models revealed only limited and mostly inconsistent evidence for such patterns. In other words, the limited, weak influence of prior awareness on PEI views and the moderately strong influence of trust in the medico-scientific establishment on several PEI views did not vary considerably across stakeholders, PEI modalities, or depression severity.

Several studies have noted the paucity of valid and reliable measures to assess knowledge of and attitudes toward PEIs^2,18,53^. Moreover, studies focusing on newer PEIs (such as rTMS) often rely on adaptations of existing ECT-related measures whose psychometric properties may not translate^2,10^. A particular strength of this study was the development of a validated and reliable measure for gathering data across multiple PEI modalities. Another strength of the current study was its survey of a large national sample of stakeholders in the United States representing key players in the use of PEIs, using a large, diverse internet panel matching current U.S. adult population estimates on several important characteristics.

Survey questions were written to ensure they were interpreted the same way across stakeholder groups; however, a limitation of the current study may be the possibility that for patients with severe depression, the vignette might not have seemed as realistic as for the other stakeholder groups. Another limitation of the current study was its narrow focus (e.g., only four PEIs were included in the study) and limited demographic factors for analysis (e.g., exclusion of U.S. census region, rural/urban area, mental and physical comorbidities, etc.). An additional limitation may be the lack of Bonferroni correction; however, Bonferroni correction was not included in statistical analysis in order to balance the benefits of increasing the likelihood of a Type I error versus a Type II error.

### Supplementary information

Supplementary Information

## Data Availability

The datasets used and/or analyzed during the current study are available from the research team upon reasonable request (lcabrera@psu.edu or mccright@msu.edu). Once we have completed the analysis of the full datasets from which this paper is part of we will post the direct links to the data repositories to https://peiproject.com/. The expected timeline for when data will be uploaded to the data repository is by the end of 2023.
