# Supplementary material for: The influence of prior awareness on views about psychiatric electroceutical interventions among non-clinician stakeholders
Source: Npj Ment Health Res. 2023 May 3;2:6. doi: 10.1038/s44184-023-00028-9 (PMC10955864; doi:10.1038/s44184-023-00028-9)
Supplement: Supplementary file 1 — Supplementary Information [file 44184_2023_28_MOESM1_ESM.pdf]

The Influence of Prior Awareness on Views about Psychiatric Electroceutical Interventions among Non-Clinician Stakeholders

Supplementary Information

Table of Contents | Page

Table of Contents	Page
Supplementary Methods	1
Supplementary Table A	8
Supplementary Table B	9
Supplementary Table C	10
Supplementary Discussion	11

Supplementary Methods

National Survey on PEI Views

[Information in brackets is not presented to participants. Unless otherwise noted, all three stakeholder groups received the same questions. Caregivers, Patients, Public denotes questions asked of these three stakeholder groups, respectively. PEI denotes where the name of a participant's assigned PEI is piped into later survey text.]

Research Participant Information and Consent Form

1. EXPLANATION OF THE RESEARCH and WHAT YOU WILL DO
You are being asked to participate in a research project that will ask you about your views about clinical depression and its treatment. After answering a few preliminary questions, you will watch a brief video showing a hypothetical interaction between a psychiatrist and patient. You will then answer a few questions about the video before answering some other questions about the proposed intervention featured in the video. At the end, you will answer some basic questions about yourself (age, education, etc.). Completing this survey should take approximately 18-20 minutes. You must be at least 18 years old to participate in this research.

2. YOUR RIGHTS TO PARTICIPATE, SAY NO, OR WITHDRAW
Participation in this research project is completely voluntary. You have the right to say no. You may also change your mind at any time and stop answering questions or skip a question if you are uncomfortable with any question.

3. COSTS AND COMPENSATION FOR BEING IN THE STUDY
You will not incur any costs for participation in this research. For your participation, you will be compensated through your regular contract with your panel provider.

4. CONTACT INFORMATION FOR QUESTIONS AND CONCERNS
If you have concerns or questions about this study, please contact the researcher by postal mail: [redacted], or by phone at [redacted], or by e-mail: [redacted].

5. CONSENT TO PARTICIPATE
By clicking on the following button, you indicate your voluntary agreement to participate in this online survey.
- ☐ I agree to participate.

When you have completed answering the question on each page, click on the right arrow at the bottom of the page to advance.

Your Caregiving Experience

[Caregivers, Public] Have you received a CLINICAL DIAGNOSIS of any of the following mental health disorders? Select all that apply.
- ☐ bipolar disorder
- ☐ clinical depression [drop respondent from the survey]
- ☐ generalized anxiety disorder
- ☐ obsessive compulsive disorder
- ☐ post-traumatic stress disorder
- ☐ I have never been diagnosed with a mental health disorder

[Caregivers, Public] In the last TWO YEARS, have you been the PRIMARY CAREGIVER for a family member, spouse, or close friend with clinical depression?
By "care," we mean helping them with daily activities, driving them to appointments, offering emotional support, etc.
- ☐ No, I have no family member, spouse, or close friend with clinical depression. [skip to first question in the next section]
- ☐ No, I have a family member, spouse, or close friend with clinical depression, but I did not provide care for them. [skip to first question in the next section]
- ☐ Yes, I have provided care for a family member, spouse, or close friend with clinical depression. [continue to the next two questions in this section]

[Caregivers] How are you related to this person?
- ☐ immediate family (parent, child, sibling)
- ☐ extended family (cousin, grandparent, in-law)
- ☐ spouse
- ☐ close friend
- ☐ neighbor, co-worker, or acquaintance

[Caregivers] Over the last TWO YEARS, what kinds of care have you provided REGULARLY? Select all that apply.

- ☐ offering emotional support via talking, texting, and e-mailing
- ☐ running errands around town
- ☐ cleaning inside or outside of home
- ☐ preparing meals
- ☐ taking care of pets or companion animals
- ☐ providing financial support
- ☐ driving them to medical appointments

Your Views on Potential Sources of Mental Health Information

Where have you gotten information about mental health in the LAST 12 MONTHS? Select all that apply.

- ☐ my primary care physician
- ☐ psychiatrists
- ☐ psychologists or other mental health care providers
- ☐ newspapers or newsmagazines
- ☐ movies or television shows
- ☐ websites or social media
- ☐ family members or friends
- ☐ scientific articles or books

How much do you DISTRUST or TRUST each of the following groups or organizations when it comes to information about your mental health?

	strongly distrust	moderately distrust	slightly distrust	neither distrust nor trust	slightly trust	moderately trust	strongly trust
my primary care physician	☐	☐	☐	☐	☐	☐	☐
psychiatrists	☐	☐	☐	☐	☐	☐	☐
the scientific community	☐	☐	☐	☐	☐	☐	☐
the US Centers for Disease Control and Prevention or CDC	☐	☐	☐	☐	☐	☐	☐
the US Food and Drug Administration or FDA	☐	☐	☐	☐	☐	☐	☐
policy-makers in elected office	☐	☐	☐	☐	☐	☐	☐
pharmaceutical companies	☐	☐	☐	☐	☐	☐	☐
medical device companies	☐	☐	☐	☐	☐	☐	☐
alternative health care providers (including naturopathic or chiropractors)	☐	☐	☐	☐	☐	☐	☐
family members or friends	☐	☐	☐	☐	☐	☐	☐
religious leaders and organizations	☐	☐	☐	☐	☐	☐	☐

A Short Video on Clinical Depression and Its Treatment

We would like you to watch a short video featuring a HYPOTHETICAL INTERACTION between a psychiatrist and her patient with treatment-resistant depression, a type of clinical depression that is rather difficult to treat.
This video is a core element of our research study.
Thus, it is important to us that you CAREFULLY WATCH this hypothetical scenario IN ITS ENTIRETY.

If you are completing our survey on your smartphone, you may need to turn your phone 90 degrees (to landscape orientation) to see the full screen.

On subsequent pages, we will ask you several questions about the proposed intervention that is featured in the video.
[one of eight randomly assigned embedded videos]
Thanks for carefully watching this hypothetical scenario, which is central to our research study.

A Few Questions about the Hypothetical Interaction in the Video

How many trials of antidepressants has Mary tried so far?

- ☐ none, only psychotherapy
- ☐ one trial of antidepressants
- ☐ two trials of antidepressants
- ☐ three trials of antidepressants

Given what you saw in the video, rate Mary's clinical depression on the following scale.

mild depression 1	2	3	moderate depression 4	5	6	severe depression 7
☐	☐	☐	☐	☐	☐	☐

What BEST describes the essence of the new intervention that Dr. Wilson suggested to Mary?

- ☐ the intervention works via ingested mood-altering chemicals
- ☐ the intervention sends electrical stimulation through the scalp
- ☐ the intervention sends magnetic stimulation through the scalp
- ☐ the intervention sends constant electrical stimulation through a brain implant
- ☐ the intervention sends variable electrical stimulation through a brain implant

Using the scales below, tell us what you think of the new intervention that Dr. Wilson suggested to Mary.

On some smartphones, you may need to scroll left and right to see the full horizontal scale.

In your opinion, [PEI] seems:

dangerous	☐	☐	☐	☐	☐	☐	☐	safe
scary	☐	☐	☐	☐	☐	☐	☐	comforting
inhumane	☐	☐	☐	☐	☐	☐	☐	humane
crude	☐	☐	☐	☐	☐	☐	☐	sophisticated
disgusting	☐	☐	☐	☐	☐	☐	☐	pleasant
barbaric	☐	☐	☐	☐	☐	☐	☐	civilized
dubious	☐	☐	☐	☐	☐	☐	☐	reputable
unpredictable	☐	☐	☐	☐	☐	☐	☐	predictable

Some Questions about [PEI]

We will now ask you some questions about the proposed intervention featured in the video. For each question on this page, place yourself in Mary's shoes and think about the intervention that Dr. Wilson proposed.
We want to know YOUR VIEWS AND ATTITUDES about this intervention.
In your view, how much would getting [PEI] INTERFERE with the following aspects of Mary's life?

	not at all interfere	minimally interfere	somewhat interfere	moderately interfere	substantially interfere	greatly interfere
the physical structure of her brain	☐	☐	☐	☐	☐	☐
the electrical signals in her brain	☐	☐	☐	☐	☐	☐
her bodily functioning in general	☐	☐	☐	☐	☐	☐
her sense of self	☐	☐	☐	☐	☐	☐
her expression of emotions	☐	☐	☐	☐	☐	☐
her daily lifestyle	☐	☐	☐	☐	☐	☐

In your view, how much of a NEGATIVE or POSITIVE influence would getting [PEI] have on the following?

	strong negative influence	moderate negative influence	slight negative influence	no influence at all	slight positive influence	moderate positive influence	strong positive influence
Mary's agency or free will	☐	☐	☐	☐	☐	☐	☐
Mary's authentic self	☐	☐	☐	☐	☐	☐	☐
Mary's ability to function in daily life	☐	☐	☐	☐	☐	☐	☐
Mary's independence	☐	☐	☐	☐	☐	☐	☐
Mary's self-control	☐	☐	☐	☐	☐	☐	☐
Mary's influence over her life circumstances	☐	☐	☐	☐	☐	☐	☐
Mary's personality	☐	☐	☐	☐	☐	☐	☐

In your view, to what extent would each of the following be a RISK for Mary getting [PEI]?

	no risk at all	minimal risk	slight risk	moderate risk	substantial risk	great risk
it causes physical injury (e.g., brain damage)	☐	☐	☐	☐	☐	☐
it creates biochemical dependence (e.g., increased craving/tolerance)	☐	☐	☐	☐	☐	☐
it triggers personality change (e.g., erratic moods)	☐	☐	☐	☐	☐	☐
it produces cognitive impairment (e.g., memory loss)	☐	☐	☐	☐	☐	☐
it provokes social stigma (e.g., negative peer judgments)	☐	☐	☐	☐	☐	☐

In your view, to what extent would each of the following be a BENEFIT for Mary getting [PEI]

	no benefit at all	minimal benefit	slight benefit	moderate benefit	substantial benefit	great benefit
it precisely targets depression in Mary's brain	☐	☐	☐	☐	☐	☐
it is safer than taking antidepressant medications	☐	☐	☐	☐	☐	☐
it quickly relieves depression symptoms	☐	☐	☐	☐	☐	☐
its stimulus is not addictive	☐	☐	☐	☐	☐	☐
its stimulus is easily adjustable	☐	☐	☐	☐	☐	☐
it reduces Mary's need to take a daily antidepressant drug	☐	☐	☐	☐	☐	☐
with it, Mary won't have to talk about her feelings	☐	☐	☐	☐	☐	☐

Some More Questions about [PEI]

Use the following scale. In your view, how bad would it be to live with treatment-resistant depression every day?

moderately bad									extremely bad
1	2	3	4	5	6	7	8	9	10
☐	☐	☐	☐	☐	☐	☐	☐	☐	☐

Think about the symptoms of treatment-resistant depression and what you now know about [PEI]. Which phrase below BEST captures your current thinking on these?

Overall, I think [PEI] seems ________ than living with treatment-resistant depression.

- ☐ much worse than
- ☐ slightly worse than
- ☐ the same as
- ☐ slightly better than
- ☐ much better than

In the comment box below, please FULLY EXPLAIN your answer to the PREVIOUS QUESTION.
The more details that you provide, the better we will understand your reasoning--which is crucial for our research project.
[insert comment box]

If you were in Mary's shoes, how UNLIKELY or LIKELY would you be to get [PEI] to improve your depression?

- ☐ very unlikely
- ☐ somewhat unlikely
- ☐ I'm not sure
- ☐ somewhat likely
- ☐ very likely

A Few More Questions about [PEI]

Consider each of the following policies or actions about using [PEI] to treat clinical depression. Indicate whether you OPPOSE or SUPPORT each one IN YOUR STATE.

	strongly oppose	moderately oppose	slightly oppose	neither oppose nor support	slightly support	moderately support	strongly support
use state tax revenue to cover the costs of using [PEI] for treating depression for those with verified financial need	☐	☐	☐	☐	☐	☐	☐
use state tax revenue to fund more research on using [PEI] for treating depression	☐	☐	☐	☐	☐	☐	☐
require each county to have at least one medical facility that offers [PEI] for treating depression	☐	☐	☐	☐	☐	☐	☐
prohibit the use of [PEI] for treating depression until we have more evidence of its safety and efficacy	☐	☐	☐	☐	☐	☐	☐
prohibit the use of [PEI] for treating depression in legal minors (under the age of 18)	☐	☐	☐	☐	☐	☐	☐
ban all use of [PEI] for treating depression	☐	☐	☐	☐	☐	☐	☐

Below are potential factors that may limit the use of [PEI] for addressing treatment-resistant depression in society generally. Please indicate what you think are the THREE MOST IMPORTANT PRACTICAL BARRIERS to its use.

Enter "1" to the left of the factor you think is the **first** most important barrier.
Enter "2" to the left of the factor you think is the **second** most important barrier.
Enter "3" to the left of the factor you think is the **third** most important barrier.

___ limited evidence of the treatment's effectiveness
___ lack of understanding of [PEI]
___ out-of-pocket cost
___ lack of insurance coverage
___ low public trust in mental health system
___ treatment is not available in all geographic areas
___ stigma about treatment
___ frequency of treatment

Below are other potential concerns you may have about [PEI] for addressing treatment-resistant depression. Please indicate your THREE TOP CONCERNS about its use.

Enter "1" to the left of the item that is your **first** most important concern.
Enter "2" to the left of the item that is your **second** most important concern.
Enter "3" to the left of the item that is your **third** most important concern.

___ limited evidence of the treatment's safety
___ treatment is too intrusive
___ patient may lack sufficient information for informed consent
___ treatment may be delivered without the patient's consent
___ patient not getting the treatment when it would actually help them

Please Tell Us about Yourself

Before today, were you aware of any of the following? Select all that apply.

☐ an adaptive brain implant
☐ antidepressant medication
☐ deep brain stimulation
☐ electroconvulsive therapy
☐ psychotherapy
☐ repetitive transcranial magnetic stimulation
☐ vagus nerve stimulation
☐ I was unaware of all of these

[Patients] Have you tried any of following interventions as a way to deal with depression? Select all that apply.

- ☐ CBD oil
- ☐ deep brain stimulation
- ☐ electroconvulsive therapy
- ☐ exercise
- ☐ ketamine
- ☐ light therapy
- ☐ repetitive transcranial magnetic stimulation
- ☐ prescription medication
- ☐ psychotherapy
- ☐ vagus nerve stimulation
- ☐ I have not tried any of these

What is your gender?

- ☐ male
- ☐ female
- ☐ non-binary
- ☐ prefer to self-describe: ___________

How old are you as of today?

- ☐ 18-24
- ☐ 25-34
- ☐ 35-44
- ☐ 45-54
- ☐ 55-64
- ☐ 65 or older

Are you Hispanic, Latino, or Chicano?

- ☐ no
- ☐ yes

What is your race/ethnicity? Select all that apply.

- ☐ White
- ☐ Black or African-American
- ☐ Native Hawaiian or Other Pacific Islander
- ☐ Native American/American Indian or Alaska Native
- ☐ Asian or Asian-American
- ☐ Arab-American or Middle Eastern
- ☐ other (please specify): ___________

What is the highest degree or credential you have earned?

- ☐ 12th grade or less
- ☐ high school diploma or GED equivalent
- ☐ associate's degree
- ☐ bachelor's degree
- ☐ master's degree
- ☐ professional degree (e.g., law or medicine)
- ☐ doctorate degree

[Caregivers, Public] Information about income is very important to understand. Would you please give your best guess? Please indicate the answer that includes your entire household income (previous year) before taxes.

- ☐ less than $25,000
- ☐ $25,000 to $49,999
- ☐ $50,000 to $74,999
- ☐ $75,000 to $99,999
- ☐ $100,000 to $149,999
- ☐ $150,000 to $199,999
- ☐ more than $200,000

Do you think of yourself as liberal or conservative?
- very liberal
- liberal
- slightly liberal
- middle-of-the-road
- slightly conservative
- conservative
- very conservative

How often do you attend religious services?
- never
- about once a year
- a few times a year
- once a month
- a few times a month
- every week
- more than once a week

Thank You!

Thank you for participating in our study. Please let us tell you more about it.

We are investigating people's views on a class of interventions for treatment-resistant depression that are called psychiatric electroceutical interventions (PEIs). Specifically, we are examining how the severity of a patient's depression and the characteristics of a PEI may influence people's views about such an intervention.

We created eight videos that featured a hypothetical interaction between a psychiatrist and her patient. Each character was played by a professional actor. [redacted] was the psychiatrist "Dr. Erica Wilson," and [redacted] was the patient "Mary." Sue Way recorded and edited the videos.

You were randomly assigned to watch one of these eight video vignettes. Your video vignette highlighted one of four PEIs: electroconvulsive therapy (ECT), repetitive transcranial magnetic stimulation (rTMS), deep brain stimulation (DBS), or an adaptive brain implant (ABI). Also, your video vignette presented a patient with either moderate or severe depression.

ECT and rTMS are approved by the US Food and Drug Administration (FDA) as therapies for depression. DBS and ABIs are still under investigation and are not FDA-approved therapies for depression.

Your participation in this study is really important to us, and your answers will help advance our understanding of how people view these types of interventions for treatment-resistant depression.

Thank you!

Supplementary Table A: Structural Factor Loadings for 32 Survey Items Measuring PEI Views in the Pooled Sample (N=3098)

**Survey Items**	**Affect toward PEI (α=0.93)**	**Perceived Influence of PEI (α=0.94)**	**Perceived Benefit of PEI (α=0.87)**	**Perceived Risk of PEI (α=0.87)**	**Perceived Invasiveness of PEI (α=0.90)**
[PEI] is dangerous to safe	**0.86**	0.36	0.29	-0.28	-0.19
[PEI] is scary to comforting	**0.78**	0.28	0.18	-0.15	-0.12
[PEI] is inhumane to humane	**0.84**	0.45	0.41	-0.35	-0.22
[PEI] is crude to sophisticated	**0.78**	0.43	0.40	-0.32	-0.21
[PEI] is disgusting to pleasant	**0.81**	0.39	0.33	-0.20	-0.13
[PEI] is barbaric to civilized	**0.84**	0.44	0.40	-0.30	-0.18
[PEI] is dubious to reputable	**0.81**	0.42	0.36	-0.24	-0.16
[PEI] is unpredictable to predictable	**0.79**	0.34	0.27	-0.18	-0.14
Mary's agency or free will	0.42	**0.85**	0.43	-0.17	-0.09
Mary's authentic self	0.44	**0.86**	0.46	-0.23	-0.13
Mary's ability to function in daily life	0.38	**0.87**	0.50	-0.24	-0.15
Mary's independence	0.40	**0.85**	0.48	-0.22	-0.15
Mary's self-control	0.41	**0.85**	0.46	-0.16	-0.07
Mary's influence over my life circumstances	0.38	**0.87**	0.50	-0.24	-0.14
Mary's personality	0.38	**0.86**	0.49	-0.21	-0.11
[PEI] precisely targets depression in Mary's brain	0.29	0.49	**0.85**	-0.15	-0.06
[PEI] is safer than taking antidepressant medications	0.42	0.39	**0.73**	-0.04	-0.05
[PEI] quickly relieves depression symptoms	0.28	0.44	**0.83**	-0.06	-0.01
[PEI]'s stimulus is not addictive	0.28	0.42	**0.79**	-0.20	-0.13
[PEI]'s stimulus is easily adjustable	0.39	0.46	**0.80**	-0.09	-0.04
[PEI] reduces Mary's need to take a daily antidepressant drug	0.26	0.44	**0.79**	-0.16	-0.07
[PEI] causes physical injury (e.g., brain damage)	-0.33	-0.21	-0.10	**0.81**	0.51
[PEI] creates biochemical dependence (e.g., increased craving or tolerance)	-0.15	-0.16	-0.13	**0.80**	0.50
[PEI] triggers personality change (e.g., erratic moods)	-0.26	-0.19	-0.09	**0.83**	0.54
[PEI] produces cognitive impairment (e.g., memory loss)	-0.28	-0.23	-0.14	**0.84**	0.47
[PEI] provokes social stigma (e.g., negative peer judgments)	-0.21	-0.18	-0.12	**0.77**	0.43
[PEI] interferes with the physical structure of her brain	-0.19	-0.21	-0.13	0.55	**0.77**
[PEI] interferes with the electrical signals in her brain	-0.22	-0.07	0.02	0.38	**0.73**
[PEI] interferes with her bodily functioning in general	-0.16	-0.18	-0.14	0.56	**0.83**
[PEI] interferes with her sense of self	-0.13	-0.10	-0.06	0.47	**0.83**
[PEI] interferes with her expression of emotions	-0.12	-0.07	-0.03	0.47	**0.83**
[PEI] interferes with her daily lifestyle	-0.15	-0.12	-0.07	0.47	**0.83**

Notes: Extraction Method: Principal Component Analysis. Rotation Method: Promax with Kaiser Normalization.

Supplementary Table B: Results of One-Way ANOVA Models Explaining Prior Awareness of Depression Interventions, Sources of Mental Health Information, and Trust in Mental Health Information Sources by Non-Clinician Stakeholder Groups

	Stakeholder Group							
	General Public		Caregivers		Depressed Patients			
Independent Variables	Mean	SD	Mean	SD	Mean	SD	F-Statistic [2, 3095]	P-Value
Pre-Survey Awareness of Depression Interventions								
antidepressant medication	0.73	0.44	0.79	0.41	0.84	0.37	17.27	<0.001
psychotherapy	0.68	0.47	0.76	0.43	0.75	0.44	7.60	<0.001
electroconvulsive therapy	0.29	0.45	0.37	0.48	0.40	0.49	14.46	<0.001
deep brain stimulation	0.14	0.35	0.24	0.43	0.20	0.40	16.47	<0.001
vagus nerve stimulation	0.07	0.25	0.14	0.34	0.13	0.34	16.60	<0.001
repetitive transcranial magnetic stimulation	0.05	0.22	0.11	0.31	0.09	0.28	11.54	<0.001
adaptive brain implant	0.07	0.26	0.10	0.31	0.09	0.29	3.24	0.039
Source of Mental Health Information								
websites or social media	0.49	0.50	0.58	0.49	0.49	0.50	11.85	<0.001
psychologists or other mental healthcare providers	0.18	0.38	0.57	0.50	0.43	0.50	190.52	<0.001
primary care physician	0.27	0.44	0.53	0.50	0.49	0.50	89.23	<0.001
family members or friends	0.30	0.46	0.45	0.50	0.32	0.47	32.75	<0.001
psychiatrists	0.10	0.31	0.41	0.49	0.30	0.46	132.83	<0.001
newspapers or newsmagazines	0.18	0.39	0.17	0.38	0.16	0.37	0.96	0.382
movies or television shows	0.22	0.41	0.15	0.35	0.18	0.38	8.60	<0.001
scientific articles or books	0.18	0.39	0.35	0.48	0.18	0.39	52.21	<0.001
Trust Moderately or Strongly in Selected Mental Health Information Sources								
my primary care physician	0.71	0.46	0.77	0.42	0.73	0.44	6.64	<0.001
psychiatrists	0.55	0.50	0.66	0.47	0.63	0.48	14.71	<0.001
scientific community	0.55	0.50	0.62	0.49	0.55	0.50	7.16	<0.001
US Centers for Disease Control & Prevention	0.52	0.50	0.56	0.50	0.54	0.50	2.37	0.094
family members or friends	0.46	0.50	0.56	0.50	0.50	0.50	11.99	<0.001
US Food & Drug Administration	0.38	0.48	0.44	0.50	0.38	0.49	4.54	0.011
alternative healthcare providers	0.24	0.43	0.37	0.48	0.28	0.45	21.16	<0.001
religious leaders & organizations	0.21	0.41	0.30	0.46	0.23	0.42	11.08	<0.001
pharmaceutical companies	0.17	0.37	0.29	0.45	0.23	0.42	21.89	<0.001
medical device companies	0.18	0.39	0.29	0.45	0.23	0.42	15.76	<0.001
policy-makers in elected office	0.10	0.30	0.15	0.36	0.14	0.34	6.41	0.002

Supplementary Table C: Standardized Coefficients from Multiple OLS Regression Models Explaining PEI Views among Non-Clinicians (N=3098)

Independent Variables	Affect toward PEI	Perceived Influence of PEI	Perceived Benefit of PEI	Perceived Risk of PEI	Perceived Invasiveness of PEI
Stakeholders					
caregivers (ref: public)	.05**	.03	.05*	.03	.04
patients (ref: public)	.06**	.04*	.05*	.02	.01
Experimental Conditions					
rTMS (ref: ECT)	.16***	.09***	.08***	-.14***	-.05*
DBS (ref: ECT)	.01	.06**	.05*	.03	.06*
ABI (ref: ECT)	-.01	.06**	.04	.06**	.07**
severe TRD (ref: moderate)	.04*	.07***	.03	.02	.03
Perception of TRD					
bad daily life with TRD	-.04*	.08***	.16***	-.03	-.02
Key Mental Health Views					
trust in medico-scientific establishment	.19***	.24***	.22***	-.01	.04*
prior PEI awareness	.10***	.03	.02	.03	.02
Socio-Demographics					
female	-.09***	.02	-.03	-.09***	-.07***
age	-.05*	-.02	-.04*	-.23***	-.21***
white	.02	.05**	.02	-.06**	-.04*
educational attainment	.10***	.04*	.03	.00	.02
political ideology	-.02	-.02	-.01	-.01	-.02
religiosity	.10***	.05*	.07***	.06**	.10***
Interaction Terms					
caregiver * prior awareness	.01	.02	.01	-.02	.02
patient * prior awareness	.02	.03	.02	.01	.01
rTMS * prior awareness	.04	.04	.04*	.00	-.00
DBS * prior awareness	.06**	.01	.04*	.02	.02
ABI * prior awareness	.06**	.00	.02	.04*	.05*
severe * prior awareness	-.02	-.01	-.01	.00	-.01
caregiver * trust in MSE	.03	.03	.04	.02	.05*
patient * trust in MSE	-.01	.02	.01	.02	.05*
rTMS * trust in MSE	-.03	.01	-.01	-.00	-.01
DBS * trust in MSE	-.00	-.00	.00	.02	-.01
ABI * trust in MSE	-.00	.01	.02	-.01	-.03
severe * trust in MSE	.02	.02	.03	.00	.01
**Adjusted R^2**	.13	.09	.10	.09	.08

* p<.05 ** p<.01 *** p<.001

Supplementary Discussion

Performance of the Demographic, Social, and Political Control Variables in Explaining PEI Views among Non-Clinicians

Female participants report less positive affect toward their assigned PEI than did their male counterparts. This parallels Naesstrom's (2017) finding that males were significantly more positive than were females about using DBS to treat obsessive compulsive disorder. Despite their male counterparts expressing more positive emotions, female participants in our study nevertheless perceive their assigned PEI to be less invasive and less risky than do the males in our study. More research on these gendered patterns will help us more fully understand the role that gender may play in non-clinicians' perception of PEIs. While little research in this area examines potential racial differences in PEI views, we do find that White adults perceived their assigned PEI slightly more positively than did their non-White counterparts. Compared to the latter, the former perceived their assigned PEI to (a) have slightly more positive influence on their self and (b) have lesser risk.

The existing literature tends to find that age is either unrelated to PEI views (e.g., Alexander 2020a; Naesstrom 2017; Tang 2002) or that age has an inconsistent relationship with PEI views (e.g., Lauber 2005; Teh 2007). Our results offer confirmation to this latter pattern. Briefly, younger adults in our study reported both more positive PEI views (i.e., more positive affect and more perceived benefit) and more negative PEI views (i.e., more perceived risk and more perceived invasiveness) than did their older counterparts.

As might be expected, education (measured as highest degree attained) related directly to affect and perceived influence. That is, more highly educated adults reported more positive affect toward—and more positive influence on self from—their assigned PEI than did their lesser educated counterparts. Further, even during our current era of heightened political polarization on many issues within the US adult population, we nevertheless found no substantial variation in PEI views across the political ideological spectrum.

Finally, our study appears to be the first to examine how religiosity (i.e., how religious someone is, independent of their religious affiliation) may relate to PEI views. Previous studies explored how religion affects perception of mental illness and treatment (Al-Natour et al. 2021; Davenport and McClure 2021; Gwin et al. 2019; Pickard 2006; Turner et al. 2018). We found, quite surprisingly, that religiosity relates positively with each of our five PEI views. Briefly, more religious adults reported both more positive PEI views (i.e., more positive affect, more perceived positive influence on the self, and more perceived benefit) and more negative PEI views (i.e., more perceived risk and more perceived invasiveness) than did their less religious counterparts. Given such seemingly inconsistent results, further research is needed on the relationships between religiosity (as well as religious affiliation) and PEI views.

Supplementary References

Al-Natour, A., Abuhammad, S., & Al-Modallal, H. Religiosity and stigma toward patients with mental illness among undergraduate university students. *Heliyon* **7**, e06565 (2021). https://doi.org/10.1016/j.heliyon.2021.e06565

Alexander, L. *et al.* Assessing public attitudes to electroconvulsive therapy: Validation of the modified ECT attitudes questionnaire using a systematic analysis. *J ECT* **36**, 47-53 (2020). https://doi.org/10.1097/yct.0000000000000612

Davenport, A. D. & McClintock, H. F. Religiosity and attitudes toward treatment for mental health in the black church. *Race Soc Probl* **13**, 226-233. http://dx.doi.org/10.1007/s12552-020-09311-2

Gwin, S. *et al.* Associations between depressive symptoms and religiosity in young adults. *J Relig Health* **59**,3193-3210 (2020). https://doi.org/10.1007/s10943-019-00889-5

Lauber, C., Nordt, C., Falcato, L. & Rössler, W. Can a seizure help? The public's attitude toward electroconvulsive therapy. *Psychiatry Res* **134**, 205-209 (2005).

Naesström, M., Blomstedt, P., Hariz, M. & Bodlund, O. Deep brain stimulation for obsessive-compulsive disorder: Knowledge and concerns among psychiatrists, psychotherapists and patients. *Surg Neurol Int* **8**, 298 (2017). https://doi.org:10.4103/sni.sni_19_17

Pickard, J. G. The relationship of religiosity to older adults' mental health service use. *Aging Ment Health* **10**, 290-297 (2006). https://doi.org/10.1080/13607860500409641

Tang, W. K., Ungvari, G. S. & Chan, G. W. Patients' and their relatives' knowledge of, experience with, attitude toward, and satisfaction with electroconvulsive therapy in Hong Kong, China. *J ECT* **18**, 207-212 (2002). https://doi.org:10.1097/00124509-200212000-00008

Teh, S. P., Helmes, E. & Drake, D. G. A Western Australian survey on public attitudes toward and knowledge of electroconvulsive therapy. *Int J Soc Psychiatry* **53**, 247-273 (2007). https://doi.org:10.1177/0020764006074522

Turner, N., Hastings, J. F., & Neighbors, H. W. Mental health care treatment seeking among African Americans and Caribbean Blacks: What is the role of religiosity/spirituality? *Aging Ment Health* **23**, 905-911. https://doi.org/10.1080/13607863.2018.1453484
